# Advanced thyroid carcinomas: neural network analysis of ultrasonographic characteristics

**DOI:** 10.1186/s13044-021-00107-z

**Published:** 2021-06-29

**Authors:** Michael Cordes, Theresa Ida Götz, Elmar Wolfgang Lang, Stephan Coerper, Torsten Kuwert, Christian Schmidkonz

**Affiliations:** 1 Radiologisch-Nuklearmedizinisches Zentrum, Martin-Richter-Str. 43, 90489 Nürnberg, Germany; 2 Nuklearmedizinische Klinik, Universitätsklinikum Erlangen, Erlangen, Germany; 3 CIML Group, Biophysics, University of Regensburg, Regensburg, Germany; 4 Klinik für Allgemein- und Viszeralchirurgie, Krankenhaus Martha-Maria, Nürnberg, Germany

**Keywords:** Advanced thyroid carcinoma, Ultrasound, Neural network, Deep learning, Artificial intelligence

## Abstract

**Background:**

Ultrasound is the first-line imaging modality for detection and classification of thyroid nodules. Certain characteristics observable by ultrasound have recently been identified that may indicate malignancy. This retrospective cohort study was conducted to test the hypothesis that advanced thyroid carcinomas show distinctive clinical and sonographic characteristics. Using a neural network model as proof of concept, nine clinical/sonographic features served as input.

**Methods:**

All 96 study enrollees had histologically confirmed thyroid carcinomas, categorized (*n* = 32, each) as follows: group 1, advanced carcinoma (ADV) marked by local invasion or distant metastasis; group 2, non-advanced papillary carcinoma (PTC); or group 3, non-advanced follicular carcinoma (FTC). Preoperative ultrasound profiles were obtained via standardized protocols. The neural network had nine input neurons and one hidden layer.

**Results:**

Mean age and the number of male patients in group 1 were significantly higher compared with groups 2 (*p* = 0.005) or 3 (*p* <  0.001). On ultrasound, tumors of larger volume and irregular shape were observed significantly more often in group 1 compared with groups 2 (*p* <  0.001) or 3 (*p* ≤ 0.01). Network accuracy in discriminating advanced vs. non-advanced tumors was 84.4% (95% confidence interval [CI]: 75.5–91), with positive and negative predictive values of 87.1% (95% CI: 70.2–96.4) and 92.3% (95% CI: 83.0–97.5), respectively.

**Conclusions:**

Our study has shown some evidence that advanced thyroid tumors demonstrate distinctive clinical and sonographic characteristics. Further prospective investigations with larger numbers of patients and multicenter design should be carried out to show whether a neural network incorporating these features may be an asset, helping to classify malignancies of the thyroid gland.

## Background

Recent investigations have shown that tumor-specific survival in patients with thyroid carcinomas is a function of various parameters [1–4], such as tumor size, extrathyroidal invasion, and distant metastasis [5, 6]. Despite the excellent 10-year overall survival (OS) rates reported for papillary (97%) and follicular (89%) carcinomas [7], invasion or distant spread of tumor does not bode well prognostically [8]. There is also substantial heterogeneity among individual tumors [9]. Genetic mutations and aggressive histotypes weigh heavily on the clinical course of thyroid cancer [10]. Results of a recently published study indicate a significantly higher prevalence of specific genetic mutations in tumors with aggressive histologic features; such mutations have been strongly linked to extrathyroidal extension or metastatic disease [11].

The American Thyroid Association guidelines delineate a comprehensive risk stratification system for patients with thyroid carcinomas [12], focusing on the presence of structurally identifiable disease after initial therapy. As stated, patients with thyroid neoplasms that show gross extrathyroidal extension (T4) or distant metastases (M1) are considered to have advanced thyroid tumors and thus represent a high-risk group. A compelling demonstration of the relationship between tumor size and invasiveness or distant spread has similarly been shown by others through multiple database (*N* = 18) compilation [13].

Ultrasound examination of the thyroid gland is the first-line imaging modality for detecting and classifying thyroid nodules. Certain features readily reflect malignancy (odds ratios of ~ 1.8–36) [14], but none are entirely specific. Current investigations have yielded some evidence that papillary (PTCs) and follicular (FTCs) carcinomas of the thyroid may differ in ultrasound features. For instance, FTCs generally surpass PTCs in terms of preoperative tumor volume, whereas the taller-than-wide (TTW) sign is typical of PTCs [15]. However, sonographic hallmarks of advanced thyroid tumors have not been consistently defined as yet. Recently, computer-aided diagnostic (CAD) systems have been tested for accuracy in interpreting thyroid nodules [16]. Those driven by neural networks may facilitate accurate disease classification by reducing complex imaging information [17].

In the present investigation, using a neural network model for proof of concept, we tested the hypothesis that advanced thyroid carcinomas (vs. non-advanced, differentiated tumors) exhibit distinctive features on ultrasound. The parameters selected were identified by cervical ultrasound examinations.

## Materials and methods

This retrospective cohort study adhered to principles of the Declaration of Helsinki and its subsequent amendments as well as guidelines of the Institutional Review Board (IRB) of the Friedrich-Alexander-University, Erlangen/Nuremberg, Germany under auspices of the Bavarian Hospital Act (Bayerisches Krankenhausgesetz Art. 27 (4)). All patients granted general permission for scientific use of their clinical data, supplying written informed consent for anonymous data publication.

**Fig. 1:**
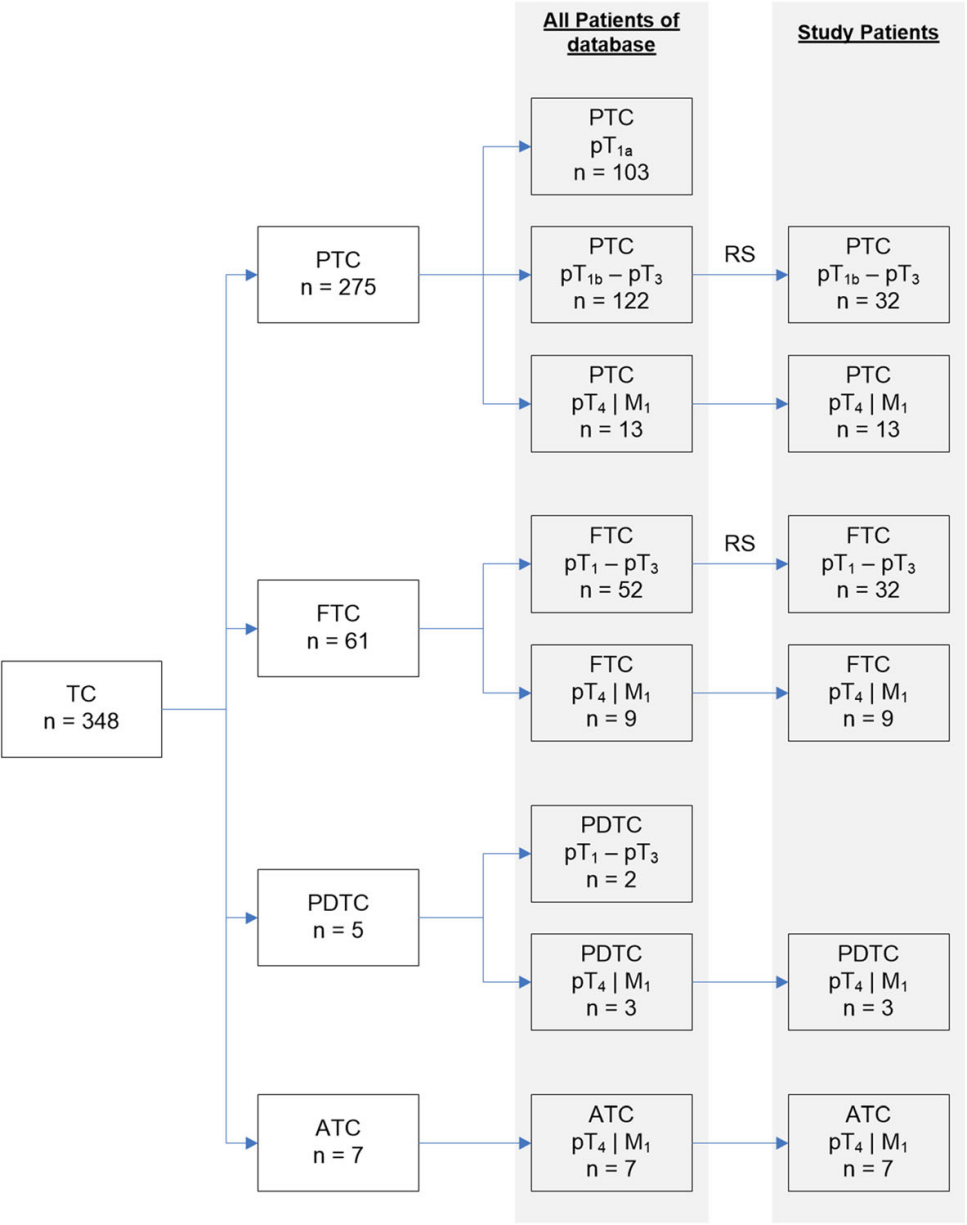
Flow chart showing the recruitment of the study individuals. The number in parenthesis represents the number of patients. (TC: thyroid carcinoma, PTC: papillary thyroid carcinoma, FTC: follicular thyroid carcinoma, PDTC: poorly differentiated thyroid carcinoma, ATC: anaplastic thyroid carcinoma, pT1–4: histological tumor stage, M1: distant metastasis, RS: random selection)

A total of 96 patients (30 men, 66 women) treated for thyroid cancer during a 10-year period (2010–2020) were enrolled for study, categorized (*n* = 32, each) as follows: group 1, advanced carcinoma (ADV: 13 men, 19 women); group 2, non-advanced PTC (6 men, 26 women); or group 3, non-advanced FTC (11 men, 21 women). ADV was defined as T4 or M1 disease stage according to the 2017 Union for International Cancer Control (UICC) TNM classification [18]. All T4 stages were confirmed by histological examination. M1 stages were assigned by imaging or histological procedures. Non-advanced tumors corresponded with stages T1–3 and M0 (no distant metastases). Equivalent patient samplings were achieved for groups 2 and 3 using a random number generator. Demographic data of all patients selected are presented in Table 1. Patients with incidental papillary microcarcinomas were ineligible to participate. The recruitment of the study subjects is shown in Fig. 1.

**Table 1:** Biographic data of the patients

	ADV (*n* = 32)	PTC (*n* = 32)	FTC (*n* = 32)	Total (*n* = 96)	***p***-value
Mean age^a^ [years]	62.8	48.9	54.5	55.4	= 0,005
SD	18.0	16.0	14.9	17.3	
Range	28–93	16–82	23–87	16–93	
m/f^b^ [n]	13/19	6/26	11/21	30/66	< 0,001

*ADV* Advanced thyroid carcinoma (group 1), *PTC* Papillary thyroid carcinoma (group 2), *FTC* Follicular thyroid carcinoma (group 3), *SD* Standard deviation, *m* Male, *f* Female. ^a^ANOVA, ^b^chi-square test

Each subject underwent thyroidectomy in one of two surgical departments. All diagnoses of thyroid carcinoma were confirmed histologically by board-certified pathologists with expertise in thyroid neoplasms. Twenty-one of the 32 patients in group 1 harbored distant metastases, present as tracer-positive lesions on whole-body iodine scans (lungs, 17; brain, 1) or identifiable by biopsy (bones, 4). This group included patients with anaplastic (ATCs, *n* = 7) and poorly differentiated (PDTCs, *n* = 3) thyroid carcinomas of follicular (*n* = 9) or papillary type (*n* = 13).

Ultrasound devices used for preoperative examinations in all patients were equipped with high-resolution longitudinal probes transmitting at a frequency of 10.0 MHz (LOGIQ P6 Pro, GE Healthcare, Chicago, IL, USA). Collected imaging data were stored in a picture archiving and communication system (PACS) for later analysis by two nuclear medicine specialists, each with more than 10 years of experience reviewing more than 2000 thyroid ultrasound examinations per year in this field.

The patients were examined in a supine position with the neck slightly extended. In this position, the anterior and lateral areas of the neck were freely accessible by the ultrasound probe. First, the complete right lobe was examined in transverse and longitudinal orientations. Second, this procedure was identically applied to the left lobe. Third, the isthmus was scanned in transverse and longitudinal orientations.

Focal lesions of the thyroid gland were recorded in two dimensions and stored to a PACS unit for later analysis. Seven morphologic tumor criteria were assessed by the examiners: (1) Volume, calculated as v = 0.5 * (dx * dy * dz) using maximum lateral (dx), anteroposterior (dy), and craniocaudal (dz) axial diameters and expressed in mL; (2) Shape, whether round (dx = dy = dz. [± 10%]), oval (> 10% disparity in axial diameters, except TTW), irregular (undulating or complex shape), or TTW (anteroposterior diameter > lateral diameter, craniocaudal diameter disregarded); (3) Contour (smooth, spiculated, or indistinctly delineated); (4) Internal structure (homogeneous vs. non-homogeneous); (5) Echogenicity, whether hypoechogenic (less than adjacent tissue but not anechoic), hypoechogenic with cysts (anechoic components), hyperechogenic (more than adjacent tissue), or hyperechogenic with cysts; (6) Calcification (+/−); and (7) Focality (one or multiple sites).

The ultrasound characteristics of the focal lesions (2) to (7) were classified according to the criteria by Russ et al. [19].

### Neural network architecture

This study was intended to distinguish advanced thyroid carcinoma from more limited forms (PTC, FTC), based on neural network processing of sonographic traits. Only one hidden layer was involved given the relative paucity of data. Demographic (age and sex) and morphologic characteristics (diameter [dx], shape, contour, structure, echogenicity, calcifications, and focality) were selected for input.

**Fig. 2:**
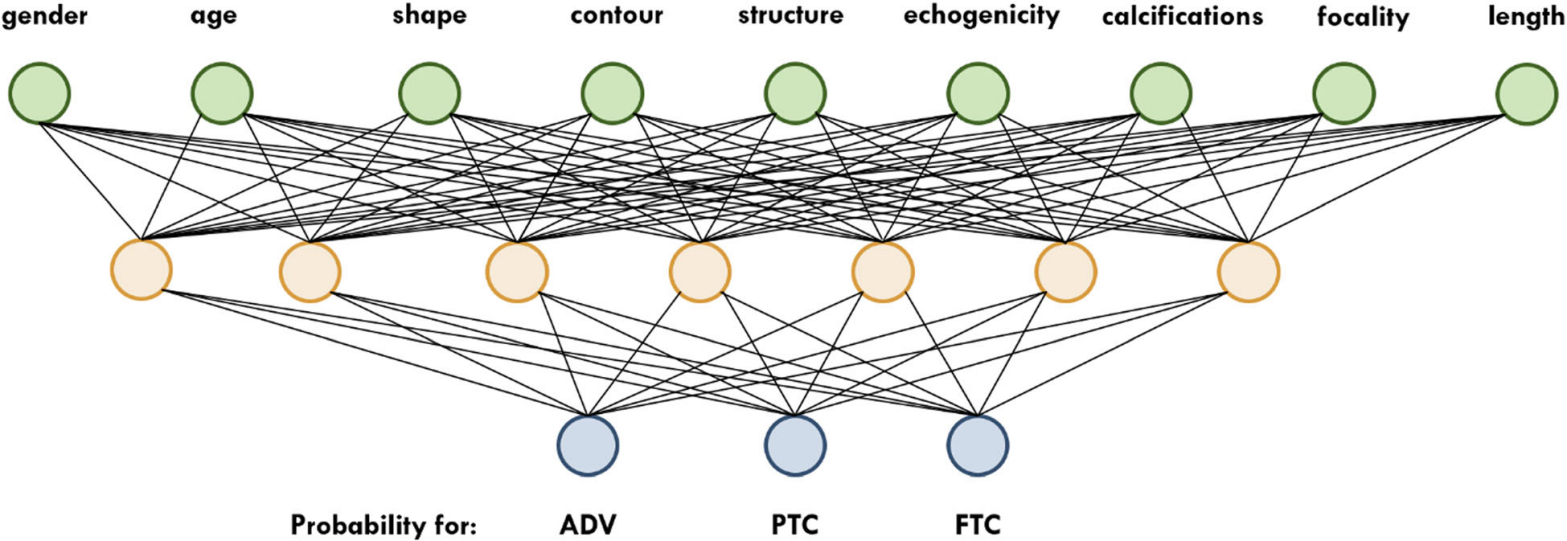
Architecture of the neural network with nine input neurons and three outputs

The network architecture is illustrated (Fig. 2). There were nine input neurons fully connected to seven hidden neurons. Output was shown as a vector indicating respective tumor probabilities. In the hidden layer, a rectified linear unit was invoked as activation function, the output layer adopting a sigmoidal function and mean squared error serving as loss function. To evaluate the network, 81 of the 96 datasets were initially used for training, reserving six for validation and nine for testing. A leave-one-out cross-validation was then carried out. The features of the implemented neural network are listed in Table 2.

**Table 2:** Features of the implemented neural network

Layer	Shape^a^	Number of Parameters	Activation Function
Input layer	(9,1)	0	–
Gated recurrent layer	[(9,7), (7)]	210	ReLU
Gated recurrent layer	[(9,3), (3)]	108	softmax
Output	(3,1)	0	–

Shape^a^: the numbers represent the data vectors, *ReLU* Rectified linear unit

### Statistical analysis

Depending on the nature of data distribution, analysis of variance (ANOVA), Fisher’s, or chi-square test was applied to test differences among groups. Significance in linear relations was gauged via Pearson’s correlation coefficient, engaging multinomial logistic regression for multivariate relations. By default, confidence intervals of binary variables involved binomial distributions. In neural network performance analysis, the following metrics were generated: accuracy, sensitivity, specificity, positive predictive value, negative predictive value, Fleiss κ, Cohens κ, and F-score. All computations were driven by standard software (MATLAB vR2012b; The MathWorks Inc., Natick, MA, USA), setting significance *p* <  0.05.

## Results

### Patient age and sex distributions

Mean age differed significantly (*p* = 0.005, ANOVA) in the three tumor subsets (ADV, 62.8 ± 18.0 years; PTC, 48.9 ± 16.0 years; FTC, 54.5 ± 14.9 years), as did male/female distributions (ADV: 41% men, 59% women; PTC: 19% men, 81% women; FTC: 34% men, 66% women; *p* <  0.001, chi-square test).

### Ultrasonographic characteristics of tumor subsets

**Fig. 3:**
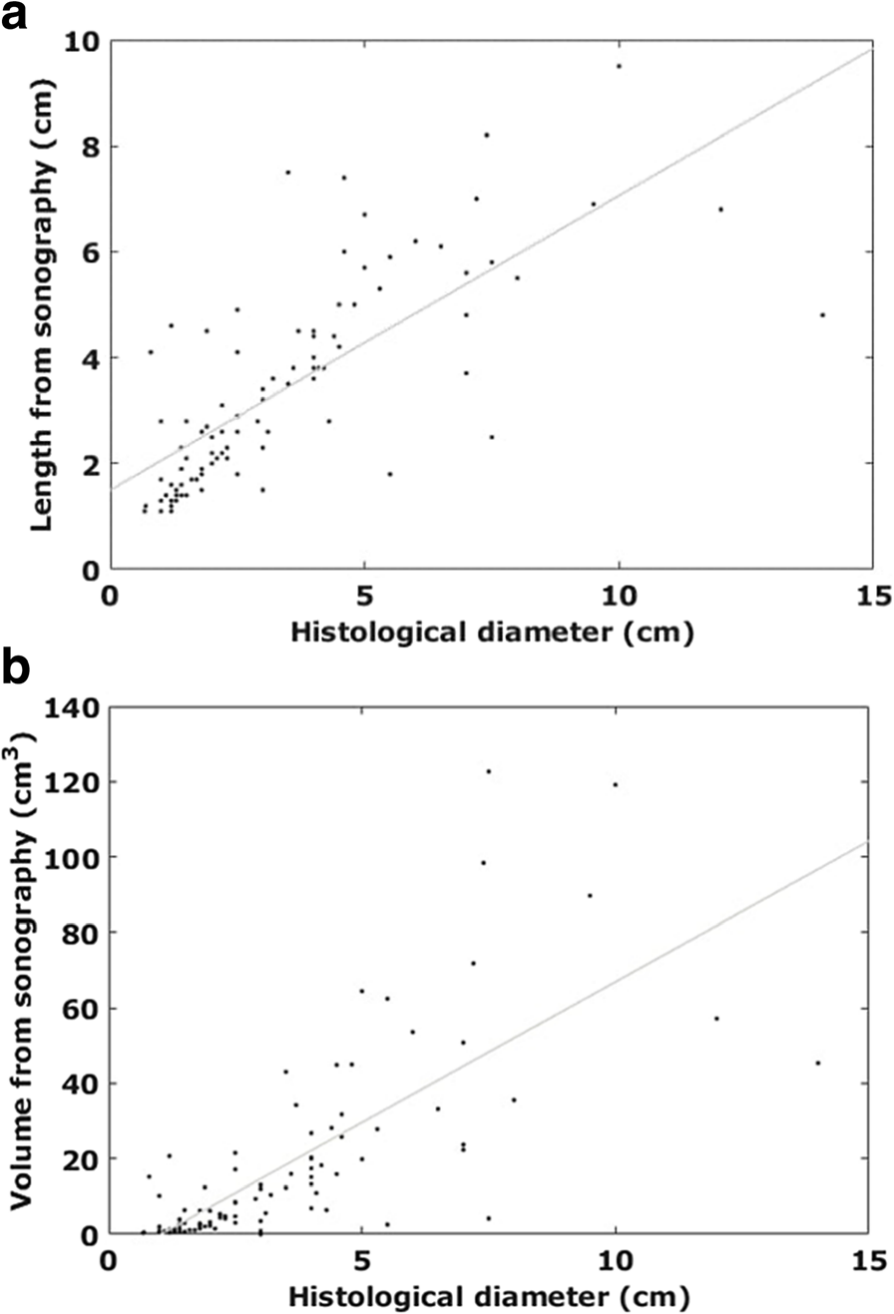
Correlation of histological diameter. **a** with sonographical length. **b** with sonographical volumetry

Average tumor size and volume obtained by ultrasound studies differed significantly (*p* <  0.001, ANOVA) among groups, determined as follows: ADV (size, 4.75 ± 2.07 cm; volume, 30.30 ± 30.68 cm^3^); PTC (size, 1.95 ± 0.88 cm; volume, 3.92 ± 8.15 cm^3^); and FTC (size, 3.58 ± 1.41 cm; volume, 19.63 ± 23.98 cm^3^). Maximum tumor diameters recorded during pathologic assessments averaged 5.45 ± 2.99 cm for ADVs, 1.72 ± 0.84 cm for PTCs, and 3.15 ± 1.59 cm for FTCs. In the entire dataset and in tumor groups, maximum tumor diameter correlated significantly with sonographic determinations of tumor size (*r* = 0.74; *p* <  0.01) and volume (*r* = 0.75; *p* <  0.01) (see Table 3, Fig. 3).

**Table 3:** Pearson correlation coefficients

	ADV (*n* = 32)	PTC (*n* = 32)	FTC (*n* = 32)	Total (*n* = 96)
Histological diameter – tumor volume				
r	0.59	0.62	0.67	0.75
p	< 0.001	< 0.001	< 0.001	< 0.001
histological diameter – tumor length				
r	0.67	0.64	0.77	0.74
p	< 0.001	< 0.001	< 0.001	< 0.001

*ADV* Advanced thyroid carcinoma (group 1), *PTC* Papillary thyroid carcinoma (group 2), *FTC* Follicular thyroid carcinoma (group 3). r: correlation coefficient, *p* p-value

Specific tumor characteristics (shape, contour, structure, echogenicity, calcifications, and focality) by ultrasound were recorded for each lesion. Feature distributions for tumor subsets (ADV, PTC, or FTC) and related statistical differences are shown in Table 4.

**Table 4:** Number of ultrasound characteristics (percent in brackets) in the study groups

Ultrasound feature	ADV (*n* = 32)	PTC (*n* = 32)	FTC (*n* = 32)	*p*-values		
				ADV vs. PTC	ADV vs. FTC	PTC vs. FTC
Shape^a^				0.004	0.010	0.002
Round	4 (13)	10 (31)	6 (19)			
Oval	4 (13)	8 (25)	23 (72)			
Irregular	24 (75)	9 (28)	2 (6)			
TTW	0 (0)	4 (13)	1 (3)			
Contour^a^				0.131	0.012	0.318
Well-defined	4 (13)	10 (31)	14 (44)			
Ill-defined	28 (88)	22 (69)	18 (56)			
Structure^a^				0.724	0.753	0.509
Homogeneous	5 (16)	4 (13)	7 (22)			
Inhomogeneous	27 (84)	28 (88)	25 (78)			
Echogenicity^a^				0.192	0.336	0.704
Hypoechogenic	26 (81)	21 (66)	21 (66)			
Hyperechogenic	5 (16)	6 (19)	8 (25)			
Cystic	1 (3)	5 (16)	3 (9)			
Calcifications^a^				0.044	0.572	0.005
Present	10 (31)	19 (59)	7 (22)			
Not present	22 (69)	13 (41)	25 (78)			
Focality^a^				0.184	0.613	0.026
Unifocal	29 (91)	24 (75)	31 (97)			
Multifocal	3 (9)	8 (25)	1 (3)			

*ADV* Advanced thyroid carcinoma (group 1), *PTC* Papillary thyroid carcinoma (group 2), *FTC* Follicular thyroid carcinoma (group 3). ^a^chi-square test

ADV, PTC, and FTC tumors were classified as EU-TIRADS V lesions in 97, 84 and 56% of cases, respectively and as EU-TIRADS IV lesions in 3, 16 and 44%, respectively. The difference between EU-TIRADS V and IV lesions was statistically significant (*p* <  0.001, chi-square test).

ln(P(t = ADV) / P(t = PTC, FTC)) = − 2.8 + 0.6 shape + 1.2 con + 0.3 struc – 0.5 echo – 0.7 calc – 0.5 foc.

To avoid one-dimensional statistical analysis, multinomial logistic regression was carried out using the following function:

Ultrasound parameters were thus used to calculate ADV probabilities, both tumor shape and contour constituting significant influences (*p* <  0.05). ADV probability was 3.6-fold greater in tumors with irregular (vs. round) shapes and increased by a factor of 3.3 if contours were irregular rather than well-defined.

### Neural network performance

In evaluating the neural network, 81 of the 96 patient datasets were initially used for training, reserving six for validation and nine for testing. Care was taken to ensure that each group had the same number of data records for the three tumor groups. Of the nine test datasets, three ADVs were clearly identified by neural network with > 90% probability, whereas PTCs were identified in only one of three instances, two improperly classified as FTCs. FTCs were identified in two of three instances, the third designated PTC.

Once the network architecture was optimized, leave-one-out validation was conducted to test its performance in tumor classification. Ultimately, 84.4% (95% confidence interval [CI]: 75.5–91) accuracy was achieved in discriminating advanced carcinomas from the other tumor subsets, with positive and negative predictive values of 87.1% (95% CI: 70.2–96.4) and 92.3% (95% CI: 83.0–97.5), respectively. Performance data for all tumor variants are shown in Table 5 and Table 6.

**Table 5:** Performance data of the neural network model for the classification of the study groups

	Total (*n* = 96)	ADV (*n* = 32)	PTC (*n* = 32)	FTC (*n* = 32)
Accuracy (95% CI)	84.4 (75.5–91.0)			
Sensitivity (95% CI)		84.4 (67.2–94.7)	87.5 (71.0–96.5)	78.1 (60.0–90.7)
Specificity (95% CI)		93.8 (84.8–98.3)	92.2 (82.7–97.4)	90.6 (79.7–96.5)
Positive predictive value (95% CI)		87.1 (70.2–96.4)	84.9 (68.1–94.9)	80.6 (62.5–92.6)
Negative predictive value (95% CI)		92.3 (83.0–97.5)	93.6 (84.5–98.2)	89.2 (79.1–95.6)
Fleiss Kappa (95% CI)	0.56 (0.55–0.57)			
F-score	0.84			

*ADV* Advanced thyroid carcinoma (group 1), *PTC* Papillary thyroid carcinoma (group 2), FTC: follicular thyroid carcinoma (group 3), CI: confidence interval

**Table 6:** Performance data of the neural network model for the classification of advanced and non-advanced thyroid carcinomas

	Total (*n* = 96)	ADV (*n* = 32)	Non-ADV (*n* = 64)	
Accuracy (95% CI)	84.4 (75.5–91.0)			
Sensitivity (95% CI)		84.4 (67.2–94.7)	82.8 (71.3–91.1)	
Specificity (95% CI)		93.8 (84.8–98.3)	83.1 (71.7–91.2)	
Positive predictive value (95% CI)		87.1 (70.2–96.4)	82.8 (71.3–91.1)	
Negative predictive value (95% CI)		92.3 (83.0–97.5)	95.3 (88.4–98.7)	
Cohens Kappa (95% CI)	0.84 (71.2–97.5)			
F-score	0.84			

*ADV* Advanced thyroid carcinoma (group 1), *Non-ADV* Non-advanced thyroid carcinoma (group 2 and 3), *CI* Confidence interval

## Discussion

Recent investigations have shown that patient prognosis is comparatively worse in advanced (vs. limited) thyroid cancers [8]. Herein, we examined demographic and sonographic parameters of patients in T4 or M1 disease stages. We also evaluated patients with limited thyroid carcinomas (stage T3 or less, no distant metastases) for purposes of comparison. To avoid sampling bias, candidates with incidental papillary microcarcinomas were deemed ineligible.

In our patient population, those with advanced disease were on average older compared to others with less prolific cancers. Male patients also accounted for a higher proportion of subjects with advanced disease. Hwang et al. have likewise identified male sex as an independent risk factor for thyroid malignancy [20]. In addition, in clinicopathologic comparisons of various thyroid carcinomas, increasing median ages among patients with anaplastic, poorly differentiated, and differentiated carcinomas have been recorded [5, 21].

Regarding sonographic parameters, we found that tumor volumes in ADV group members significantly surpassed those of the limited disease groups. Overall, tumor size determined by ultrasound correlated well with measurements obtained during pathologic examination, although multinominal logistic regression analysis revealed a more than three-fold rise in the incidence of advanced (vs. limited) disease for tumors with irregular shapes and contours.

The impact of tumor size on risk of T4 disease stage or distant metastases has already been explored in an earlier study [13]. These authors found that in differentiated thyroid carcinomas, the risk of local invasion (T4) or distant spread (M1) increases gradually along with tumor size. Such increases appeared linear for PTCs (without threshold effect) and non-linear for FTCs beyond 4 cm in diameter. In terms of distant metastases, no size thresholds were evident for PTCs or FTCs, although the probability of distant metastases increased progressively with size in undifferentiated thyroid cancers.

The mean tumor diameter we determined for all types of ADVs was ~ 5.4 cm. Subgroup analysis further revealed mean tumor diameters of 1.7 cm and 3.1 cm for PTCs and FTCs, respectively. These findings imply that in the context of advanced thyroid cancers, no threshold values are definable for culpable primary tumors.

In our patients, irregularly shaped tumors were statistically more frequent in those with advanced (vs. limited) disease. A large-scale meta-analysis has also shown that irregular margins (among other features) are highly predictive of malignancy [14]. Unfortunately, the ultrasound features of advanced or non-advanced tumors were not addressed, particularly ramifications of round, oval, or irregular sonographic tumor shapes.

Hahn et al. have reported that shapes and margins of thyroid tumors on ultrasound may reflect levels of biologic aggression [22]. For instance, oval-to-round appearances and well-defined margins were detected more often in poorly differentiated carcinomas than in anaplastic tumors. These revelations perhaps support the significant disparities in irregular tumor shapes and margins exhibited by advanced and non-advanced tumors in the course of our multinomial logistic regression analysis.

Our investigation was not designed as an observer study. Therefore, we are unable to provide data on the intra- and interobserver variances of the ultrasound findings. Ultrasound examinations were carried out, and the results were classified by examiners with high experience in this field. We assume that this approach was feasible to keep the variances low.

The structure of neural networks, as well as training and validation processes, has been extensively described by Lee et al. [17]. They contend that this technology may help integrate the diagnostic intricacies of complex pathologies. Reliance on neural networks for quantitative data processing may indeed provide greater diagnostic accuracy in patients with suspected advanced cancerous lesions.

We used a two-step approach to evaluate our network. Training and validation were done alternately to ensure sufficient generalizability during training, testing predictive power on a hold-out dataset. Training was halted when the loss of function converged. Our proof-of-concept network led to correct classification in most patients (84%) with ADVs. Jeong et al. have evaluated a commercially available CAD system for ultrasonographic recognition of thyroid cancers [16], reaping a positive predictive value of 81.3%. However, these commercially available artificial intelligence systems were devoid of clinical input, restricted to ultrasound parameters only [23].

As a retrospective study, Li et al. recently examined the diagnostic performance of a deep convolutional network model to differentiate malignant and benign thyroid nodules based on ultrasound imaging data [24]. The observed accuracy of this model in correctly classifying respective lesions was also quite high (> 85%). Our smaller sampling achieved similar accuracy (84.4%) in discriminating advanced from limited thyroid cancers, thus indicating the high potential of adjunctive neural network learning methods in imaging analysis. Unlike the model of Li et al., our approach allows the implementation of a freely accessible online data input tool. Because voluminous data is not essential, our application is a practical one.

Besides the neural network that was used in our study to classify thyroid nodules, several other approaches using computer-aided diagnosis systems (CAD) have been evaluated. Wei et al. [25] compared the diagnostic value of S-Detect, a CAD system used to differentiate benign and malignant thyroid nodules by radiologists with different levels of experience. They reported that S-Detect had an accuracy, sensitivity, specificity, positive predictive value, and negative predictive value of 77.0, 91.3, 65.2, 68.3, and 90.1%, respectively. We could demonstrate a higher efficiency of our neural network approach. Furthermore, in contrast to our study, in Wei et al.’s study not all of the histopathological results were obtained by surgical resection; this might have further restricted the study results. Additionally, Kim et al. reported that S-Detect has a limitation in the evaluation of nodule calcifications, restricting its use in the evaluation of calcified thyroid nodules [26]. Xia et al. evaluated the use of S-Detect in 171 patients with 180 thyroid lesions [27]. They found that the CAD system presented a higher sensitivity but lower specificity than an experienced radiologist (90.5% vs. 81.1 and 41.2% vs. 83.5%). The radiologist also had a higher accuracy compared to the CAD system (82.2% vs. 67.2%) for diagnosing malignant thyroid nodules. The authors concluded that S-Detect had a lower specificity and accuracy than the experienced radiologist in identifying papillary thyroid carcinomas and also maintained a relatively lower performance than the experienced radiologist in identifying follicular thyroid carcinomas. Unlike S-Detect, our presented neural network approach allows the implementation of a freely accessible online data input tool that enables simple non-commercial use in the future.

There are several limitations to our study, the first being its retrospective design. We included only those thyroid tumors from our database that were identifiably encoded. Another issue is that only patients surgically treated at our facility with available pathologic reports were considered. Various protocols used were also clinically based and non-standardized, and the small number of patients involved who were not perfectly matched may have introduced significant outcome bias. Our investigation was performed as a single center retrospective study. One should be aware that this design might have reduced the statistical power of our results. A bias regarding the parameter tumor size as input function of the neural network cannot be excluded in our data. However, this parameter alone is not decisive as to whether the neural network classifies a tumor as advanced or not. In the advanced tumor group, 21 of 32 patients presented with distant metastases and were therefore included in the advanced group. Finally, we used a concise rather than comprehensive neural network model for analysis, requiring some simplification of output functions.

## Conclusion

From our study, we have found some evidence that advanced thyroid tumors show distinctive clinical and sonographic characteristics. Further prospective investigations with larger numbers of patients and multicenter design should be carried out to show whether a neural network incorporating these features may be an asset, helping to classify malignancies of the thyroid gland.

## Data Availability

Available.
